# Supplementary figures and images for: Susceptibility of Aedes albopictus, Ae. aegypti and human populations to Ross River virus in Kuala Lumpur, Malaysia
Source: PLoS Negl Trop Dis. 2023 Jun 12;17(6):e0011423. doi: 10.1371/journal.pntd.0011423 (PMC10289418; doi:10.1371/journal.pntd.0011423)

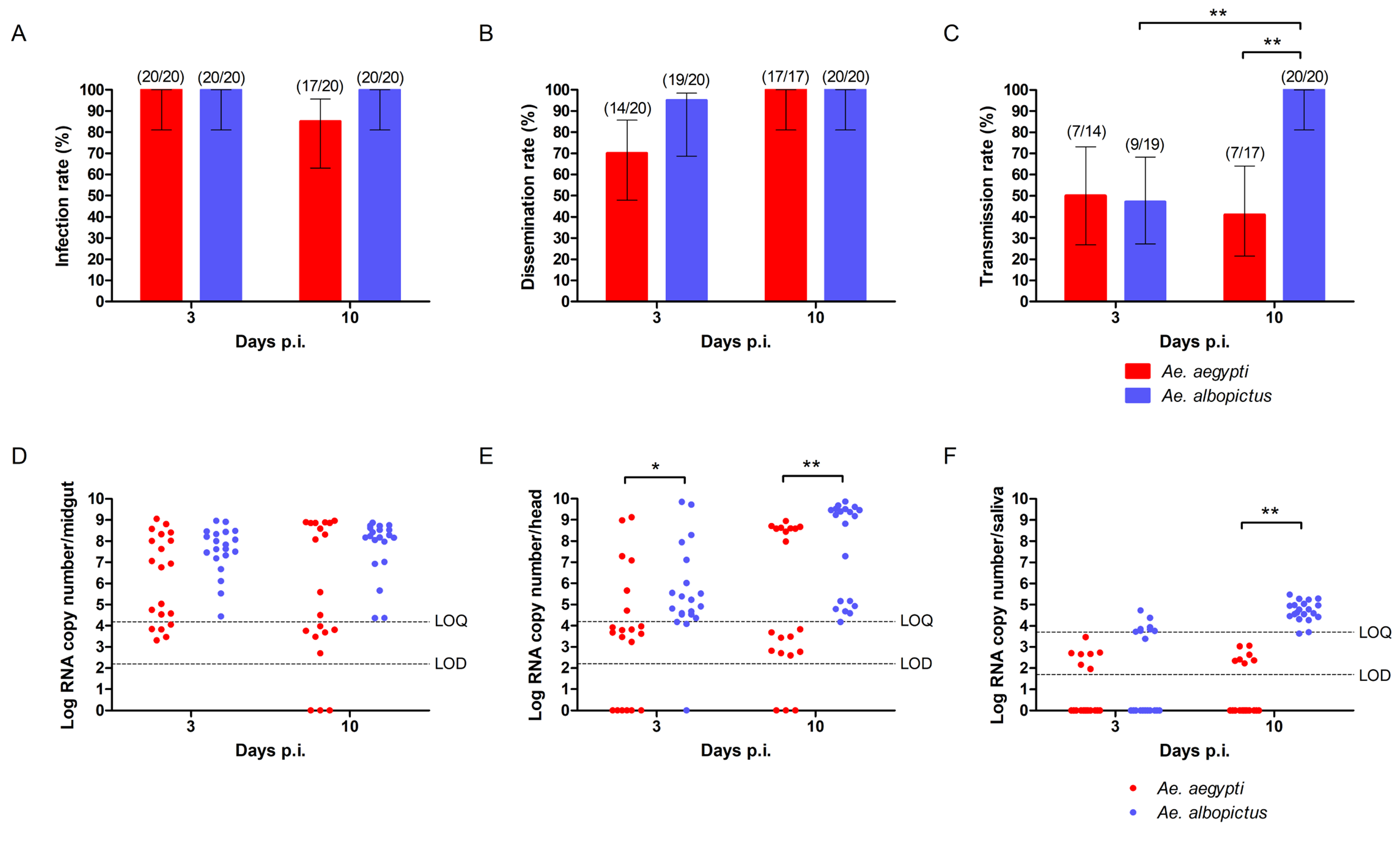

Supplement: S1 Fig — (TIF) [file pntd.0011423.s003.tif]
