# Supplementary material for: Susceptibility of Aedes albopictus, Ae. aegypti and human populations to Ross River virus in Kuala Lumpur, Malaysia
Source: PLoS Negl Trop Dis. 2023 Jun 12;17(6):e0011423. doi: 10.1371/journal.pntd.0011423 (PMC10289418; doi:10.1371/journal.pntd.0011423)
Supplement: S1 Table — (DOCX) [file pntd.0011423.s004.docx]

**S1 Table. Salivary RRV loads in *Ae. aegypti* and *Ae. albopictus* mosquitoes with detectable virus.**

| **Days post-infection** | ***Ae. aegypti*** | | | ***Ae. albopictus*** | | | **t-test (degrees of freedom)** | **P value** |
| --- | --- | --- | --- | --- | --- | --- | --- | --- |
|  | **n** | **Mean (SD) viral load** | **95% CI** | **n** | **Mean (SD) viral load** | **95% CI** |  |  |
| 3 | 7 | 2.6 (0.5) | 2.2-3.1 | 9 | 3.9 (0.4) | 3.6-4.2 | 6.0 (14) | <0.0001** |
| 10 | 7 | 2.6 (0.3) | 2.3-2.9 | 20 | 4.7 (0.5) | 4.5-4.9 | 10.5 (25) | <0.0001** |

SD, standard deviation; CI, confidence intervals.

Viral loads are measured in log_10_ RNA copies/mosquito.

Mean viral loads of the two mosquito species are compared at each time point, and significant differences are shown (unpaired t-test; *, p<0.01, **, p<0.0001).
