# Supplementary material for: Susceptibility of Aedes albopictus, Ae. aegypti and human populations to Ross River virus in Kuala Lumpur, Malaysia
Source: PLoS Negl Trop Dis. 2023 Jun 12;17(6):e0011423. doi: 10.1371/journal.pntd.0011423 (PMC10289418; doi:10.1371/journal.pntd.0011423)
Supplement: Attachment — Submitted filename: Remarks for the authors.docx [file pntd.0011423.s005.docx]

Remarks to the Authors:

General comment

The manuscript is relatively well written, and the results are interesting. However, some part of the manuscript must be rewritten especially the results and the discussion parts as described in the specific comment. Firstly, I would like to suggest being careful with the results because authors analyzed the body, head and saliva by RT-PCR not by virus detection with cells. Secondly, I really think the second part of the vector competence study must be rewritten. Indeed, authors decided to use different virus titer in the blood meal according with the previous results obtained in the manuscript. That is interesting but that also limited the comparison between both vectors. I recommend to describe this part one vector and after the other one but not to compare both in all this part. Finally, the discussion must be summarized and clarified. I think this manuscript needs major revision before being accepted for publication.

Specific comment

Abstract

1) I think the Methodology part could be summarized and clarified.

Abstract summarize

2) Line 60: some Pacific islands
3) Line 66: higher instead of greater
4) Line 68-69: This sentence is not adapted, no context no link with the previous sentence.
5) Line 70: Modulate the sentence.

Introduction

6) Line 76: Add a reference after “public health issue”.
7) Line 77: The term person-to-person is not adapted for arbovirus which need the presence of vector for their transmission.
8) Line 82: Modified as “Ross River virus (RRV; *Togaviridae* family) which ….”
9) Line 83: Add region after Pacific.
10) Line 101: Add a reference after “bitten by the mosquitoes”.
11) Line 102: Add a reference after “combinaison”.
12) Line 106: modulate the sentence
13) Line 112-118: This part is confusing and need to be clarified.

Methods

14) Line 127-131: What is the mosquito generation used for the experimental infection?
15) Line 145-174: This part is confusing, maybe that could help the readers if the two parts are separated in different sections.
16) Line 148: Did the authors use total blood or just the erythrocytes?
17) Line 210-211: Remove this sentence.
18) Line 220-242: A lot of information are missing
    a. How many sera were tested?
    b. Line 232-234: How authors determined the number of sera needed to evaluated the cross reactivity? Is it enough with 16 sera?
19) Line 240: Why did authors incubate the plates only 2 days and not 3 days as other *Togaviridae* (ex: CHIKV)
20) Line 264-269: This part must be clarified.

Results

21) Line 274-292: This part could be summarized.
22) Line 278 (at among the result part): indicate the statistical test used for this result.
23) Line 328-336: This part must be modulated due to the low number of samples for some days post-infection.
24) Line 361-432: In this part, authors decided to adapt the virus titer in the blood meal according to the first results obtained in their study. This is interesting. However I think for the results part authors could not compared *Ae. aegypti* and *Ae. albopictus* due to this difference in the infectious blood meal. They must separate the result according to the vector. And maybe at the end of each part add a sentence to indicate the differences between both vectors according to each virus titer.
25) Line 437-445: The part is not adapted in the manuscript, no link with the other ones. Maybe it could be better to place this part at the beginning of the result part as a context.

Discussion

26) Line 450: Add a reference after “Malaysia”.
27) Line 262-263: More factors are needed for the emergence and spread of an arbovirus. Add other factors.
28) Line 264-265: Remove the sentence after [28].
29) Line 467-480: This part could be summarized.
30) Line 470-472: This sentence must be modulated due to the difference in the infectious blood feeding titer.
31) Line 475: Add a reference after “short-lived”.
32) Line 476-480: This part is not clear and need to be clarified.
33) Line 482-491: This part could be summarized.
34) Line 495: Add a reference after “other arboviruses”
35) Line 498: Change “head titres”	as “virus titer in heads” or in other formulation.
36) Line 498-507: This part could be summarized.
37) Line 509-522: This part could be summarized.
38) Line 524-526: Remove this sentence.
39) Line 527-538: This part could be summarized.
40) Line 551: Add a reference after “RRV”.
41) Line 552-569: I really appreciated that the authors clearly indicated the limits of their study. I just think the authors could discuss the fact they tested only *Aedes* vectors maybe it could be interesting to test also some other mosquito species.

Figures

42) In their figure, authors presented Infection, Dissemination and Transmission rates but for me the indicators used aren’t rate but efficiency due to the fact they are calculated among the number of mosquitoes tested. Authors should modify the figure and the legend.
