# Supplementary material for: Susceptibility of Aedes albopictus, Ae. aegypti and human populations to Ross River virus in Kuala Lumpur, Malaysia
Source: PLoS Negl Trop Dis. 2023 Jun 12;17(6):e0011423. doi: 10.1371/journal.pntd.0011423 (PMC10289418; doi:10.1371/journal.pntd.0011423)
Supplement: Attachment — Submitted filename: Response to reviewers.docx [file pntd.0011423.s006.docx]

**RESPONSE TO REVIEWERS (PNTD-D-22-01316)**

We thank you and the reviewers for your comments, which we have addressed below. Our responses refer to line numbers in the revised manuscript.

**Reviewer's Responses to Questions**

**Reviewer #1: The study has met the acceptance criteria listed in Methods.**

**Reviewer #2: The technics used in this manuscript are adapted but some information are missing.**

**Authors didn't describe as they determined the number of sera used for the seroprevalence study for the cross-reactivity part.**

**Response:** We arbitrarily decided on 16 serum samples with high levels of anti-DENV and anti-CHIKV antibodies (to maximise occurrence of cross-reactivity), which we feel is sufficient.

**The analysis by RT-PCR limited the interpretation due to the detection of both dead and infectious viral particles.**

**Response:** We agree that an infectious assay would have been valuable, although sensitivity is much lower than PCR. However, we feel that presence of viral RNA in saliva, for example, is highly likely to represent shed virus from salivary glands. PCR is used to detect RRV in saliva for surveillance of field-caught mosquitoes in Australia (Hall-Mendelin *et al*., 2010). In our experience, saliva arboviral loads can be too low to detect by infectious assays. Miller *et al*. (2021) reported low ZIKV and CHIKV titres of 10-100 pfu/saliva sample in infected *Ae. aegypti*, and demonstrated transmission to highly susceptible, immunocompromised mice from infected mosquitoes with low levels of arboviral RNA and undetectable infectious virus in saliva. Thus, given the limited volumes to work with, we elected to use PCR due to higher sensitivity. We have also addressed this in the limitations paragraph in the discussion.

References

1. Hall-Mendelin S, et al. Exploiting mosquito sugar feeding to detect mosquito-borne pathogens. Proc Natl Acad Sci U S A 2010;107(25):11255-9.

2. Miller MR, et al. Characterizing and quantifying arbovirus transmission by *Aedes aegypti* using forced salivation and analysis of bloodmeals. Insects 2021;12(4):304.

**Reviewer #3: The aims of the study are clearly stated, and the methods followed are in accordance with the study objectives. They used field populations of the mosquitoes that they tested and an appropriate population size. I would prefer to have a proven vector of the virus as a positive control.**

**Response:** The objective of the study was to test if Malaysian *Aedes* mosquitoes are competent vectors for RRV, rather than compare the competence with proven vectors. Also, the common Australian vectors *Culex annulirostris*, *Ae. vigilax* and *Ae. camptorhynchus* are not available to us and are either uncommon or not reported in Malaysia.

**I also think that it would be more logical to use the same dose of the virus for the two mosquito species that were tested for the competence or use the two different doses and check the infection-dissemination-transmission-transmission efficiency for both Ae albopictus and Ae aegypti.**

**Response:** Thank you for this comment. As the OID experiment indicated large differences in OID for the two mosquito species, we used different RRV blood meal concentrations to ensure infection rates were >90% for both. This allowed comparison of vector competence parameters with a similar starting infection rate. We used 7 log_10_ PFU/ml blood meal for *Ae. aegypti* to ensure >90% infection. However, for *Ae. albopictus*, as 5 log_10_ PFU/ml blood meal was sufficient to ensure >90% infection, we felt that using 7 log_10_ PFU/ml blood meal was excessive. If our results then showed that *Ae. albopictus* had lower vector competence with the lower concentration blood meal, this would have been difficult to support. However, we consistently found that *Ae. albopictus* showed higher vector competence than *Ae. aegypti* (despite 100× lower concentration blood meal), so we feel this finding is still valid. This comparison is highly relevant in Malaysia as the rural/urban distribution is different for these two species.

**My only concern is that they didn't use an infectious assay at least for determining the presence of the infectious virus in the saliva.**

**Response:** We feel that presence of viral RNA in saliva is highly likely to represent shed virus from salivary glands. PCR is used to detect RRV in saliva for surveillance of field-caught mosquitoes in Australia (Hall-Mendelin *et al*., 2010). In our experience, saliva arboviral loads can be too low to detect by infectious assays. Miller *et al*. (2021) reported low ZIKV and CHIKV titres of 10-100 pfu/saliva sample in *Ae. aegypti*, and demonstrated transmission to highly susceptible, immunocompromised mice from infected mosquitoes with low levels of arboviral RNA and undetectable infectious virus in saliva. Thus, given the limited volumes to work with, we elected to use PCR due to higher sensitivity. We have also addressed this in the limitations paragraph in the discussion.

References

1. Hall-Mendelin S, et al. Exploiting mosquito sugar feeding to detect mosquito-borne pathogens. Proc Natl Acad Sci U S A 2010;107(25):11255-9.

2. Miller MR, et al. Characterizing and quantifying arbovirus transmission by *Aedes aegypti* using forced salivation and analysis of bloodmeals. Insects 2021;12(4):304.

**Summary and General Comments**

**Reviewer #1: The work is nicely done and presented. I am not an English language/grammar expert to make comments on its writing, but scientifically the paper is sound. Just few minor comments:**

**1. Recommend revising the title. The word "High" seems vague, may be delete this from the title and leave rest of the sentence as is.**

**Response:** We agree and have removed the word “High” from the title.

**2. Abstract/L28: Suggest changing "Worldwide" to "widely distributed in countries of tropical and subtropical regions" or sth similar. The distribution of chikungunya and Zika is not yet worldwide.**

**Response:** We have replaced the word “worldwide outbreaks” to “widespread outbreaks in tropical and subtropical regions”. Refer to line 28.

**3. L168-169: "Dissection needles were soaked in 70% alcohol between each mosquito". 70% alcohol disinfect the virus but not really destroy their nucleic acids. When was the control uninfected mosquito dissected - if control mosquito was dissected at first and test mosquitoes later on just soaking the needle in alcohol in-between of dissection, then there might be some possible contamination issues with the procedures. Soaking dissection needle in 10% bleach, then to alcohol is recommended.**

**Response:** Thank you for the comment. The whole negative control mosquito was tested without dissection. We soaked the dissection needles in 70% alcohol and thoroughly wiped dry prior to dissection (lines 175-176). We feel that the chances of contamination are minimal as there were no unexpected results (e.g. positive head with negative midgut) seen. Furthermore, as the infected mosquitoes were dissected in the sequence shown in S1 data (‘replication kinetics’ tab), we also observed several instances of a sample with high viral load followed by a negative sample (e.g. *Ae. aegypti* samples 8 and 12 at day 3), indicating no carryover. Nevertheless, we will adopt the reviewer’s recommendation for future experiments.

**Reviewer #2: The manuscript is relatively well written, and the results are interesting.**

**1. However, some part of the manuscript must be rewritten especially the results and the discussion parts as described in the specific comment. Firstly, I would like to suggest being careful with the results because authors analyzed the body, head and saliva by RT-PCR not by virus detection with cells.**

**Response:** We have addressed the reviewer’s comments on the results and discussion. We have addressed the limitation of PCR in the discussion. We feel that presence of viral RNA in saliva is highly likely to represent shed virus from salivary glands, and hence potential to transmit. PCR is used to detect RRV in saliva for surveillance of field-caught mosquitoes in Australia (Hall-Mendelin *et al*., 2010). In our experience, saliva arboviral loads can be too low to detect by infectious assays. Miller *et al*. (2021) reported low ZIKV and CHIKV titres of 10-100 pfu/saliva sample in *Ae. aegypti*, and demonstrated transmission to highly susceptible, immunocompromised mice from infected mosquitoes with low levels of arboviral RNA and undetectable infectious virus in saliva. Thus, given the limited volumes to work with, we elected to use PCR due to higher sensitivity.

**2. Secondly, I really think the second part of the vector competence study must be rewritten. Indeed, authors decided to use different virus titer in the blood meal according with the previous results obtained in the manuscript. That is interesting but that also limited the comparison between both vectors. I recommend to describe this part one vector and after the other one but not to compare both in all this part.**

**Response:** Thank you for this comment. As the OID experiment indicated large differences in OID for the two mosquitoes, we used different RRV blood meal concentrations to ensure infection rates were >90% for both. This allowed comparison of vector competence parameters with a similar starting infection rate. We used 7 log_10_ PFU/ml blood meal for *Ae. aegypti* to ensure >90% infection. However, for *Ae. albopictus*, as 5 log_10_ PFU/ml blood meal was sufficient to ensure >90% infection, we felt that using 7 log_10_ PFU/ml blood meal was excessive. If our results had shown that *Ae. albopictus* had lower vector competence with the lower concentration blood meal, this would have been difficult to support. However, we consistently found that *Ae. albopictus* showed higher vector competence than *Ae. aegypti* (despite 100× lower concentration blood meal), so we feel this finding is valid. This comparison is highly relevant in Malaysia as the rural/urban distribution is different for these two species.

Therefore, we hope to strike a compromise on this point. We have separated the vector competence results into two parts based on each vector as suggested, and revised the text and figures in this results section, entitled “Malaysian *Ae. aegypti* and *Ae. albopictus* are potential RRV vectors”. We have also included the comparison of the 2 species with a justification into a separate section entitled “*Ae. albopictus* is a more competent vector for RRV than *Ae. aegypti*”, and shifted the relevant figure and table into Supporting Information to make it less prominent. We hope that is agreeable. However, we can easily remove this separated results section and supporting figure/table if felt necessary upon further review.

**3. Finally, the discussion must be summarized and clarified. I think this manuscript needs** **major revision before being accepted for publication.**

**Response:** We have edited the discussion from 1,614 to 1,514 words.

**Reviewer #3: Overall, I think it is a good draft.**

**1. I would suggest discussing more the fact that they used only PCR for determining transmission instead of an infectious assay.**

**Response:** As mentioned earlier, this has been included and expanded in the discussion of limitations of the study.

**2. In addition, I would add something about the role that the animals play (or not) in spreading the virus and may suggest in the Conclusion if further research is needed regarding the role of animals.**

**Response:** We have included the following sentences in the discussion:

“Significant RRV outbreaks and enzootic transmission have also occurred in regions without marsupials, such as the Pacific Islands and metropolitan areas in Australia (32,33). High viraemia in human patients and low seroprevalence in nonhuman vertebrates during these outbreaks provide strong evidence for human-mosquito-human transmission (31). “

And we have added this to the concluding paragraph:

“The possible roles of animals as reservoirs should be explored, although enzootic transmission may not be critical if these *Aedes* mosquito species are involved as vectors, as they preferentially feed on humans (62).”

**3. Also, in the Conclusion part it should be addressed the fact that the sero-prevalence of the virus in human population is still low, pointing that, for now, the risk is low in Malaysia.**

**Response:** Rather than the risk being low, we feel that the low seroprevalence in humans indicates “a naive host population” (as written in the conclusion), which indicates a high potential risk of an outbreak should the virus be introduced.

**Results**

**Reviewer #2: I think this part must be rewritten.**

**Authors should be careful with the interpretation of the results due to the protocols used.**

**Response:** We have revised the results part according to reviewer #2’s earlier comment.

**Conclusions**

**Reviewer #2: The discussion must be clarified and summarized.**

**Response:** We have edited the discussion from 1,614 to 1,514 words.

**Reviewer #3: They don't have a conclusion part, but a summary as a last paragraph in Discussion. I think that they did well to mention the probable risk that the virus may pose, but the fact that they did find a very low sero-prevalence of the virus in the human population in Kuala Lumpur should be further address and maybe suggest further research regarding the role of animals in the circulation of the virus in the area.**

**Response:** This summary paragraph also includes our conclusions. The low seroprevalence of RRV in population has been discussed in lines 574-585. We have added this to the concluding paragraph:

“The possible roles of animals as reservoirs should be explored, although enzootic transmission may not be critical if these *Aedes* mosquito species are involved as vectors, as they preferentially feed on humans (62).”

**Editorial and Data Presentation Modifications?**

**Reviewer #2: Major modifications needed**

**Reviewer #3: I would suggest a minor modification to the title of the article "High susceptibility of Aedes albopictus and Ae. aegypti to Ross River virus and sero-prevalence of human population in Kuala Lumpur, Malaysia". Also in line 493: "..and (space) Ae. aegypti..."**

**Response:** Taking into account reviewer #1’s suggestion for a title change, we have settled on: “Susceptibility of *Aedes albopictus*, *Ae. aegypti* and human populations to Ross River virus in Kuala Lumpur, Malaysia”. We would prefer not to add “seroprevalence of” as we feel it leads to multiple subjects and reduces clarity of the title, but are happy to change if required.

We have added the missing gap, thank you.

**Comments from the file “Remarks for the authors”:**

**Abstract**

1) **I think the Methodology part could be summarized and clarified.**

**Response:** We have revised the methodology part.

**Abstract summarize**

2) **Line 60: some Pacific islands**

**Response:** We have changed ‘the Pacific islands’ to ‘some Pacific islands’.

3) **Line 66: higher instead of greater**

**Response:** Revised.

4) **Line 68-69: This sentence is not adapted, no context no link with the previous sentence.**

**Response:** We have revised the sentence to: “To determine population immunity against RRV in Kuala Lumpur, Malaysia, we tested 240 patient blood samples and found only 0.8% had neutralizing antibodies against RRV.”

We have also made minor changes to the rest of the Author Summary to keep the word count under 200 as per instructions to authors.

5) **Line 70: Modulate the sentence.**

**Response:** We have simplified the sentence to: “In summary, Malaysia is at risk of an RRV epidemic due to presence of a naive host population and abundant competent mosquito vectors.”

**Introduction**

6) **Line 76: Add a reference after “public health issue”.**

**Response:** Reference added.

7) **Line 77: The term person-to-person is not adapted for arbovirus which need the presence of vector for their transmission.**

**Response:** We have revised the term “person-to-person” to “virus transmission” in line 76.

8) **Line 82: Modified as “Ross River virus (RRV; *Togaviridae* family) which ….”**

**Response:** Revised.

9) **Line 83: Add region after Pacific.**

**Response:** Added.

10) **Line 101: Add a reference after “bitten by the mosquitoes”.**

**Response:** References 14 & 15 added.

11) **Line 102: Add a reference after “combination”.**

**Response:** Reference 13 added.

12) **Line 106: modulate the sentence**

**Response:** The sentence has been revised to "As RRV is able to infect multiple mosquito and vertebrate species, it has potential to establish viral transmission cycles, especially in high risk areas with abundant mosquito vectors and naive populations (16)”. Refer to lines 107-110. Reference 16 has been added.

13) **Line 112-118: This part is confusing and need to be clarified.**

**Response:** We have rewritten these sentences and hope they are now clearer.

**Methods**

14) **Line 127-131: What is the mosquito generation used for the experimental infection?**

**Response:** F3 or F4 generations of the mosquitoes were used. This has been added to line 135.

15) **Line 145-174: This part is confusing, maybe that could help the readers if the two parts are separated in different sections.**

**Response:** We have separated the section (previously “infection and harvest of mosquito”) into two parts as suggested: “Mosquito infection” (from line 151) and “Vector competence analysis” (line 167).

16) **Line 148: Did the authors use total blood or just the erythrocytes?**

**Response:** We used fresh whole blood. Refer to line 154.

17) **Line 210-211: Remove this sentence.**

**Response:** The sentence referred to (now line 216) is “An older study showed a range of RRV seroprevalence of 0-4% in non-endemic countries in Southeast Asia.” We feel this sentence is important as it justifies the figures used in the next sentence to calculate the sample size needed.

18) **Line 220-242: A lot of information are missing**
    a. **How many sera were tested?**

**Response:** Total sera tested was 240 (see line 219).

b. **Line 232-234: How authors determined the number of sera needed to evaluated** **the cross reactivity? Is it enough with 16 sera?**

**Response:** We arbitrarily decided on 16 serum samples with high levels of anti-DENV and anti-CHIKV antibodies (to maximise occurrence of cross-reactivity), which we feel is sufficient.

19) **Line 240: Why did authors incubate the plates only 2 days and not 3 days as other *Togaviridae* (ex: CHIKV)**

**Response:** The RRV neutralization assay incubation was 3 days (line 233), but the incubation for CHIKV was 2 days (line 246). This was because CHIKV grows faster and we found that the plaques were too big to count if incubated for 3 days.

20) **Line 264-269: This part must be clarified.**

**Response:** (Now referring to lines 270-274) The purpose of this section is to define "possible" and "probable" RRV-seropositive samples according to the presence of anti-CHIKV antibodies, which theoretically may cross-react and give false positive results for anti-RRV antibodies. We have rewritten it to make it clearer.

**Results**

21) **Line 274-292: This part could be summarized.**

**Response:** We have summarized the paragraph.

22) **Line 278: indicate the statistical test used for this result.**

**Response:** Added statistical test “Fisher’s exact test”.

23) **Line 328-336: This part must be modulated due to the low number of samples for some days post-infection.**

**Response:** We make the point that “although only 3 *Ae. aegypti* mosquitoes were infected at this dose” (3 log_10_ PFU/ml) in line 335. The other comparisons showed statistical differences with higher numbers of samples.

24) **Line 361-432: In this part, authors decided to adapt the virus titer in the blood meal according to the first results obtained in their study. This is interesting. However I think for the results part authors could not compared *Ae. aegypti* and *Ae. albopictus* due to this difference in the infectious blood meal. They must separate the result according to the vector. And maybe at the end of each part add a sentence to indicate the differences between both vectors according to each virus titer.**

**Response:** Thank you for this comment. As the OID experiment indicated large differences in OID for the two mosquitoes, we used different RRV blood meal concentrations to ensure infection rates were >90% for both. This allowed comparison of vector competence parameters with a similar starting infection rate. We used 7 log10 PFU/ml blood meal for *Ae. aegypti* to ensure >90% infection. However, for *Ae. albopictus*, as 5 log10 PFU/ml blood meal was sufficient to ensure >90% infection, we felt that using 7 log10 PFU/ml blood meal was excessive. If our results then showed that *Ae. albopictus* had lower vector competence with the lower concentration blood meal, this would have been difficult to support. However, we consistently found that *Ae. albopictus* showed higher vector competence than *Ae. aegypti* (despite 100× lower concentration blood meal), so we feel this finding is valid. This comparison is highly relevant in Malaysia as the rural/urban distribution is different for these two species.

Therefore, we hope to strike a compromise on this point. We have separated the vector competence results into two parts based on each vector as suggested, in a results section entitled “Malaysian *Ae. aegypti* and *Ae. albopictus* are potential RRV vectors”.

The reviewer suggests “at the end of each part add a sentence to indicate the differences between both vectors according to each virus titer.” To avoid repetition, instead of comparing the differences after each vector, we have also separated the comparison of the 2 species with a justification into a new section entitled “*Ae. albopictus* is a more competent vector for RRV than *Ae. aegypti*”, and shifted the relevant figure and table into Supporting Information to make it less prominent. We hope that is agreeable. However, we can easily remove this separated results section and supporting figure/table if felt necessary upon further review.

25) **Line 437-445: The part is not adapted in the manuscript, no link with the other ones. Maybe it could be better to place this part at the beginning of the result part as a context.**

**Response:** As mentioned in the introduction, two crucial factors governing risk of an arbovirus outbreak are presence of competent mosquitoes and susceptible human hosts. Thus, while most of the study focused on mosquito vector competence in Kuala Lumpur, an important part of the work was to determine population susceptibility to RRV if it was newly introduced - this was the basis of the seroprevalence study. We have therefore left this results section in its current position, and retitled it from "Very low RRV seroprevalence in Kuala Lumpur" to "Population susceptibility to RRV in Kuala Lumpur" to make its relevance clearer.

**Discussion**

26) **Line 450: Add a reference after “Malaysia”.**

**Response:** Reference 30 added (now line 500).

27) **Line 462-463: More factors are needed for the emergence and spread of an arbovirus. Add other factors.**

**Response:** In addition to competent vectors, we have added ideal climatic conditions and human herd immunity as additional factors needed for the emergence and spread of an arbovirus. Refer to lines 512-514.

28) **Line 264-265 (actually referring to 464-465): Remove the sentence after [28].**

**Response:** The sentence was removed.

29) **Line 467-480: This part could be summarized.**

**Response:** We have summarized this paragraph.

30) **Line 470-472: This sentence must be modulated due to the difference in the infectious blood feeding titer.**

**Response:** This comment refers to the sentence: “*Ae. albopictus* had a lower threshold for oral infection, with significantly lower OID_50_ and OID_75_ than *Ae. aegypti*.” This sentence is correct, as both mosquito species were challenged with the same blood meal titres of RRV in order to determine OID.

31) **Line 475: Add a reference after “short-lived”.**

**Response:** We have removed the sentence as part of the response to comment no. 29. Therefore, no reference is added.

32) **Line 476-480: This part is not clear and need to be clarified.**

**Response:** We have clarified this as part of the response to comment no.29. Previous lines 476-477 have been removed.

33) **Line 482-491: This part could be summarized.**

Response: We have summarized the paragraph.

34) **Line 495: Add a reference after “other arboviruses”**

**Response:** We have changed “alphavirus” to “arboviruses” (now line 540). References were added.

35) **Line 498: Change “head titres” as “virus titer in heads” or in other formulation.**

**Response:** We have changed to “head titres”	as “virus titres in heads” in line 542-543.

36) **Line 498-507: This part could be summarized.**

**Response:** We have summarized the paragraph.

37) **Line 509-522: This part could be summarized.**

**Response:** We have summarized the paragraph.

38) **Line 524-526: Remove this sentence.**

**Response:** We feel that this paragraph is important to discuss the bimodal distribution of RRV viral loads observed (and mentioned several times, e.g. in Table 2) in the results section. Therefore, we would like to maintain this sentence.

39) **Line 527-538: This part could be summarized.**

**Response:** We have summarized the paragraph.

40) **Line 551: Add a reference after “RRV”.**

**Response:** Reference 53 added.

41) **Line 552-569: I really appreciated that the authors clearly indicated the limits of their study. I just think the authors could discuss the fact they tested only *Aedes* vectors maybe it could be interesting to test also some other mosquito species.**

**Response:** We decided to test RRV competence in these two *Aedes* vectors because they are the predominant arbovirus vectors in Malaysia and easily collected from the field. We agree that testing other vectors (particularly confirmed RRV vectors) is important for future work, and have included this in the paragraph: “it would be important to test vector competence of other common *Aedes* and *Culex* species found in Malaysia” (lines 588-590).

**Figures**

42) **In their figure, authors presented Infection, Dissemination and Transmission rates but for me the indicators used aren’t rate but efficiency due to the fact they are calculated among the number of mosquitoes tested. Authors should modify the figure and the legend.**

**Response:** We have defined these rates (lines 176-180) the same way as numerous other papers (three refs below). We have added the definitions to the Figure 2 & 3 labels for clarity. For example, our “dissemination rate (number of PCR-positive heads/number of PCR-positive midguts)”, is the same calculation used by Merwaiss *et al.* (2021), which further defines dissemination efficiency as "proportion of females with infected head tissues among all tested females".

References

1. Merwaiss F, et al. Chikungunya virus replication rate determines the capacity of crossing tissue barriers in mosquitoes. J Virol 2021;95(3):e01956-20. doi: 10.1128/JVI.01956-20.

2. Tsetsarkin KA, et al. A single mutation in chikungunya virus affects vector specificity and epidemic potential. PLoS Pathog 2007;3(12):e201. doi: 10.1371/journal.ppat.0030201.

3. McGee CE, et al. Infection, dissemination, and transmission of a West Nile virus green fluorescent protein infectious clone by *Culex pipiens quinquefasciatus* mosquitoes. Vector Borne Zoonotic Dis 2010;10(3):267-74. doi: 10.1089/vbz.2009.0067.
