# Supplementary material for: Susceptibility of Aedes albopictus, Ae. aegypti and human populations to Ross River virus in Kuala Lumpur, Malaysia
Source: PLoS Negl Trop Dis. 2023 Jun 12;17(6):e0011423. doi: 10.1371/journal.pntd.0011423 (PMC10289418; doi:10.1371/journal.pntd.0011423)
Supplement: Attachment — Submitted filename: Response to reviewers.docx [file pntd.0011423.s007.docx]

Response to Editor:

We thank you and the reviewers for your comments, which we have addressed below. Our responses refer to line numbers in the revised manuscript without tracked changes.

Reviewer's Responses to Questions

**Methods

Reviewer #1: The study has met the acceptance criteria listed in Methods. The aims of the study are clearly stated, and the methods explained here address the stated objectives.**

**Reviewer #2:
I still think the number of samples tested for the seroprevalence part is not enough. However authors indicated this limitation in the discussion part.**
Response: We justified the number of samples tested in the methods (lines 213-215): “Using an estimated prevalence of 3.5% and precision of 2.5%, we calculated a minimum sample size of 208.” (We tested 240 samples.) However, we do agree that more sites should be sampled, as mentioned as a limitation.

**Reviewer #3: (No Response)**


**Results

Reviewer #1: Results are complete and clearly presented.**

**Reviewer #2: Results were well presented.
Figure 1: Error bar not visible especially for the Ae. albopictus condition.
              Legend is not complete for the A part.**

Response: Thank you, we have adjusted the colour of all error bars for Figs 1-3 to black, for consistency and clarity. We have readjusted Fig 1 to make all legends fully visible.

**Reviewer #3: (No Response)

Conclusions

Reviewer #1: Authors mentioned the probable RRV risk in Kuala Lumpur. In abstract as well as in the main text, they have toned up stating Malaysia at high risk of an imported RRV outbreak. However, their own results of sero-prevalence (RRV IgG) in local residents and unavailability of RRV's putative hosts and their vectors other than Ae. albopictus, not really suggest that Malaysia is at a high risk. Better to tone-down the conclusion.**

Response: We have noted that low seroprevalence in people means there is no underlying susceptibility, thus the population is at risk from imported virus. We have also noted the lack of putative animal hosts has not prevented RRV outbreaks from happening in other places. Nevertheless, we have revised lines 53, 106, 112, 497 to tone down the conclusion of the risk of RRV epidemic in Malaysia.

Line 51: removed “high” from “high risk”

Line 106: removed “high risk”

Line 112: “Risk” replaced with “potential”

Line 503: removed “high” from “high susceptibility”

**Reviewer #2: The discussion part could be summarized.**
Response: The discussion wordcount has been reduced by a further 10% to make it more succinct.

**Reviewer #3: (No Response)**

**Editorial and Data Presentation Modifications?**

**Reviewer #1: Accept

Reviewer #2: (No Response)

Reviewer #3: (No Response)

Summary and General Comments

Reviewer #1: Overall, the work is nicely done, and the paper is executed well. Sero-prevalence of the virus in human population not concerning that clearly suggesting that there is no circulation of RRV in the local setting of the KL. A low tone of RRV risk would better fit for the conclusion.**

Response: We have noted that low seroprevalence in people means there is no underlying susceptibility, thus the population is at risk of imported virus. Nevertheless, we have revised lines 53, 106, 112, 497 to tone down the conclusion of the risk of RRV epidemic in Malaysia.

Line 51: removed “high” from “high risk”

Line 106: removed “high risk”

Line 112: “Risk” replaced with “potential”

Line 503: removed “high” from “high susceptibility”

**Reviewer #2: Authors really improved the manuscript according to the reviewer' comments.
Minor revisions are required for acceptance.**

**Line 44-46: no link with the previous sentence**

Response: We have revised this.

**Line 52: remove "and is more likely primary vector" because that depend on the vector density also**

Response: Removed.

**Line 54: remove "high" before risk**

Response: Removed.

**Line 72: modulate the sentence**

Response: We have rearranged the sentence for clarity.

**Method: in Viruses part authors must present the DENV strain used also**

Response: Cross-neutralization assay against an RRV isolate was done using DENV-antibody positive serum and CHIKV-antibody positive serum. We did not perform neutralization of live DENV.

**Line 522 and 614: mistake in ref 6 and 60 (X))**

Response: The reviewer is referring to the use of brackets within brackets; this has been corrected (now referring to ref 6 and 59 in lines 450 and 534, respectively).

**The discussion must be summarized.**
Response: The discussion wordcount has been reduced by a further 10% to make it more succinct.

**Reviewer #3: (No Response)**
